# The Morbid Effects of the Retention in the Blood of the Elements of the Urinary Secretion

**Published:** 1861-11

**Authors:** 


Art. VIII.—*The Morbid Effects of the Retention in the Blood of the Elements of the Urinary Secretion.* By William Wallace Morland, M.D., etc. Fiske Fund Prize Essay. 8vo., pp. 83. Philadelphia: Blanchard & Lea, 1861.

This paper, which is a reprint from the *American Journal of the Medical Sciences* for April and July, 1861, investigates more fully than ever before, perhaps, a matter of experimental inquiry of a very important and interesting kind. The subject, as proposed by the Trustees of the Fiske Fund, necessitates, we are told—1st. The enumeration of "the elements of the urinary secretion." 2d. The recital of the effects produced by the undue "retention" of each of these in the blood. Under the latter category uræmia is considered in relation to pregnancy and parturition, gout, rheumatism, etc.; and each element in the composition of the urine is studied in its important pathological relations. The subject is ably discussed, and all that it has been possible for the writer to collect of information bearing upon the effects of such retentions, is given by him.

